# P-2221. Clinical utility and sensitivity of plasma metagenomic microbial cell-free DNA sequencing in children with complicated pneumonia

**DOI:** 10.1093/ofid/ofae631.2375

**Published:** 2025-01-29

**Authors:** Erin C Ho, Edwin J Asturias, Molly Butler, Dennis Simmons, Samuel R Dominguez

**Affiliations:** University of Colorado School of Medicine, Aurora, CO; CU School of Medicine, Aurora, Colorado; Children's Hospital Colorado, Aurora, Colorado; Children's Hospital Colorado, Aurora, Colorado; University of Colorado School of Medicine, Aurora, CO

## Abstract

**Background:**

Etiological diagnostics for children with complicated bacterial pneumonia remain limited and insensitive, and pleural fluid (PF) testing is invasive. Plasma metagenomic microbial cell-free DNA (mcfDNA) sequencing, available by Karius, Inc., is a potentially comprehensive, non-invasive, and efficient diagnostic tool for pediatric pneumonia. However, rigorous studies comparing the real-world performance of mcfDNA to other testing modalities are lacking.

**Figure:**
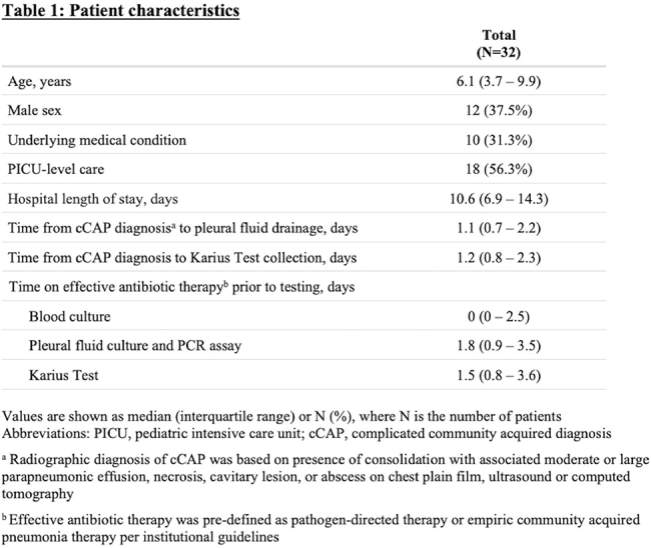

**Methods:**

We compared the performance of the Karius Test (KT) to blood culture, PF culture, and in-house PF PCR assays targeting the most common pathogens in complicated community acquired pneumonia (cCAP), including *Streptococcus pneumoniae* (Spn), group A *Streptococcus* (GAS), and *Staphylococcus aureus*, in immunocompetent children hospitalized with cCAP requiring pleural effusion or empyema drainage at Children’s Hospital Colorado from 2022-2024. We calculated percent agreement across tests and sensitivities using both a composite reference standard and clinical truth. Bacterial pathogens were adjudicated by three infectious disease physicians as probable, possible, or unlikely cCAP pathogens. DNA virus detections on KT were not considered causative of cCAP.

**Figure:**
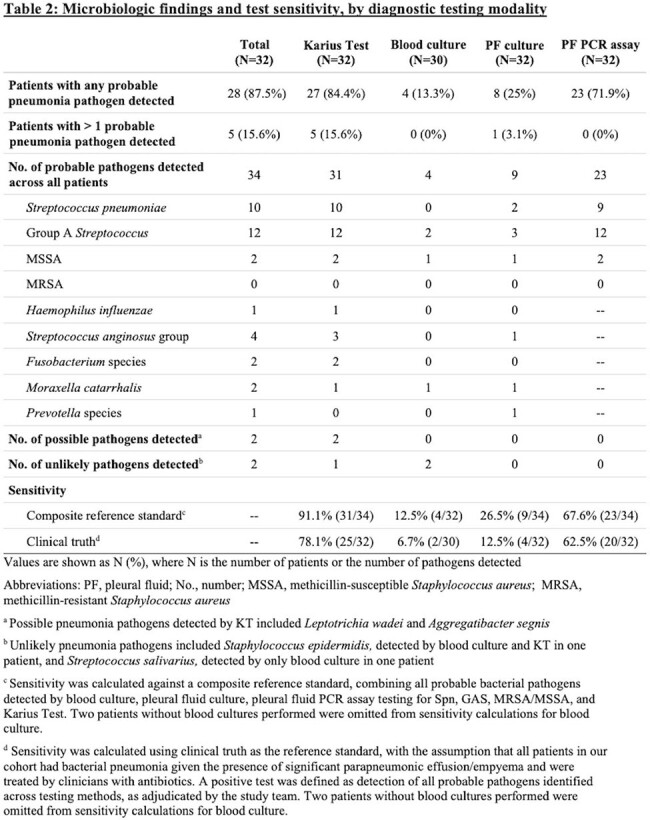

**Results:**

In our cohort of 32 children with cCAP (Table 1), 84.4% (27/32) had at least one probable pathogen identified by KT alone vs. 75% (24/32) by all other testing combined (Table 2). GAS (37.5%) and Spn (31.3%) were the most commonly detected pathogens, and 15.6% had > 1 probable pathogen detected. Compared to a composite reference standard, KT had the highest sensitivity (91.1%), followed by targeted PF PCR assays (67.6%), PF culture (26.5%), and blood culture (12.5%), with similar trends by clinical truth. Positive agreement between KT and PCR + culture was 88.9%. Most KT samples were collected within 5 days of starting effective antibiotic therapy, but KT remained positive even up to 10 days on therapy (Figure 1).

**Figure:**
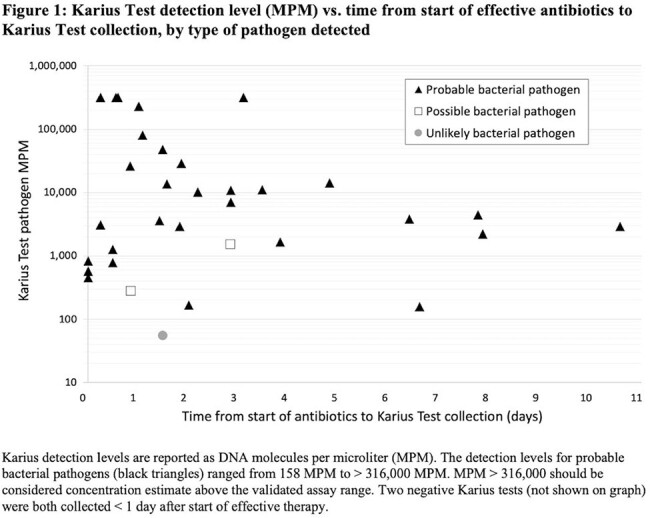

**Conclusion:**

Compared to culture and PF PCR-based testing, KT had higher sensitivity and excellent positive agreement for etiological diagnosis of pediatric cCAP. Early use of KT may be a promising diagnostic strategy, especially in the setting of antibiotic pre-treatment. Further studies are needed to determine KT specificity and clinical impact.

**Disclosures:**

Edwin J. Asturias, MD, Hillevax: Advisor/Consultant|Merck: Advisor/Consultant|Moderna: Advisor/Consultant|Pfizer: Grant/Research Support Molly Butler, PhD, Pfizer: Conference Attendance Samuel R. Dominguez, MD, PhD, BIofire Diagnostics: Advisor/Consultant|BIofire Diagnostics: Grant/Research Support|DelveBio: Grant/Research Support|Karius: Advisor/Consultant|Karius: Grant/Research Support|Pfizer: Grant/Research Support

